# Motor impairment in a rare form of spastic paraplegia (Spoan syndrome): a 10-year follow-up

**DOI:** 10.1186/s12883-019-1465-5

**Published:** 2019-10-27

**Authors:** Cláudia R. C. Galvão, Priscilla M. A. Cavalcante, Ricardo Olinda, Zodja Graciani, Mayana Zatz, Fernando Kok, Silvana Santos, Selma Lancman

**Affiliations:** 1 Department of Occupational Therapy, Federal University of Paraíba, João Pessoa, Brazil; 2 Rehabilitation Sciences Program, University of São Paulo, São Paulo, Brazil; 3 Department of Statistics, State University of Paraíba, Campina Grande, Brazil; 4 Department of Physical Therapy, Mackenzie Presbyterian University, São Paulo, Brazil; 5 Department of Genetics and Evolutionary Biology, University of São Paulo, São Paulo, Brazil; 6 Department of Neurology, University of São Paulo, São Paulo, Brazil; 7 Community Genetics Group, State University of Paraíba, Rua das Baraúnas 351, Campina Grande, Paraíba Brazil

**Keywords:** Spastic paraplegia, Optic atrophy, Rare diseases, Longitudinal survey

## Abstract

**Background:**

Spastic paraplegia, optic atrophy and neuropathy (Spoan syndrome) is an autosomal recessive disease with approximately 70 cases recorded in Brazil and Egypt.

**Methods:**

This is a prospective longitudinal study performed with 47 patients affected with Spoan syndrome of seven communities of Rio Grande do Norte (Brazil) to investigate changes in motor function based on comparative data obtained from a 10-year follow-up.

**Results:**

The mean age of the participants was 47.21 ± 12.42 years old, and the mean age at loss of ambulation and hand function were 10.78 ± 5.55 and 33.58 ± 17.47 years old, respectively. Spearman’s correlation analysis between the score on the Modified Barthel Index and the investigated variables evidenced statistical significance for age (*p* < 0.001) and right- and left-hand grip strength (*p* = 0.042 and *p* = 0.021, respectively). Statistical significance was not evidenced for the remainder of the variables, including age at onset of symptoms (*p* = 0.634), age at loss of ambulation (*p* = 0.664) and age at loss of hand function (*p* = 0.118).

**Conclusions:**

Our analysis allows asserting that the participants exhibited slight dependence until age 35. The greatest losses occurred from ages 35 to 41, and starting at 50, practically all patients become completely dependent. These findings are relevant for determining the prognosis as well as suitable treatment, rehabilitation and assistive technology for these individuals.

## Background

Spastic paraplegia, optic atrophy and neuropathy (Spoan syndrome) (OMIM #609641) is an autosomal recessive disease. Individuals with Spoan syndrome exhibit early-onset progressive spastic paraplegia, resulting in loss of independent ambulation before adolescence, combined with non-progressive congenital optic atrophy. Axonal neuropathy develops over the first decade of life and results in progressive loss of upper limb function, together with other manifestations, such as dysarthria, shortening of muscles and spine deformities [1, 2]. More than 70 cases have been recorded, two in Egypt and all others in Brazil, three in the South and Southeast regions and the remainder mainly in the Northeast [3].

The mutation involved consists of a 216-bp microdeletion in the 11q13 chromosome region, which upregulates the expression of the *KLC2* gene product, a kinesin related to axonal transport [3]. The initial diagnosis of Spoan syndrome is made in the clinic, based on neurological examination, followed by genetic testing aiming at detecting the aforementioned mutation, which is performed at the Centre of Studies of the Human Genome, University of São Paulo.

In the area known as Alto Oeste Potiguar, Rio Grande do Norte, Brazil, the prevalence of Spoan syndrome is 1:250 inhabitants, and it is estimated that 1 in 15 inhabitants are heterozygous carriers of the mutation [4]. Spoan syndrome is thus a genetic disease that causes severe physical disability and has high prevalence in the Brazilian Northeast region. However, the incidence of the disease may be underestimated globally given that some cases might not be diagnosed, as Spoan can be confounded with other simple or complex forms of spastic paraplegia [5].

The aim of this prospective longitudinal study was to investigate a rare form of spastic paraplegia (Spoan syndrome) and changes in motor function based on comparative data obtained from a 10-year follow-up of 47 patients. This data are relevant for the purpose of communicating prognosis to patients and for selecting adequate outcome measures for future clinical studies.

## Methods

The present was a prospective and longitudinal study on the motor function of individuals with confirmed diagnosis of Spoan syndrome in which data collected in 2007 and 2017 were compared. Of the 61 participants in the first assessment, 47 resided in municipalities in Alto Oeste Potiguar, Rio Grande do Norte, and could be located with the help of community health workers. These patients were assessed at home by occupational therapists.

The distribution of the patients per municipality was as follows: São Miguel (*n* = 17), Serrinha dos Pintos (*n* = 13), Pau dos Ferros (*n* = 5), Encanto (*n* = 4), Coronel João Pessoa (*n* = 3), Dr. Severiano (*n* = 3) and Martins (*n* = 2). Fourteen of the participants in the original study were excluded for the following reasons: not residing in the investigated municipalities (*n* = 6), death (*n* = 8) and lack of confirmation of Spoan syndrome on genetic testing (*n* = 1).

The 47 participants in the present study were assessed using the same protocols as the ones used by Graciani and colleagues (2010). The participants were first interviewed to collect data such as date of birth, educational level, age at onset of symptoms and ages at loss of ambulation and hand function. Next, we applied the following instruments for the measurement of motor function parameters:

1. Modified Barthel Index (MBI), described by Shah et al. (1989) [6]: this index determines the degree of dependence of individuals relative to 10 functional categories – (1) personal hygiene, (2) bathing, (3), feeding, (4) toilet use, (5) stair climbing, (6) dressing, (7) bladder control, (8) bowel control, (9) ambulation and (10) transfers. The total score ranges from 0 to 100, and the results are categorised as follows: 0–20 – total dependence; 21–60 – severe dependence; 61–90 – moderate dependence; 91–99 – slight dependence; and 100 – independence.
2. Spastic Paraplegia Rating Scale (SPRS) [7]: this instrument consists of 13 variables to measure the severity of spastic paraplegia. The individual scores of variables range from 0, normal, to 4, severe impairment. The maximum total score is 52.
3. Functional Hereditary Spastic Paraplegia Rating Scale (FHSPRS) [8]: this scale measures levels of dysfunction based on functional locomotion impairment and ambulation quality, need of auxiliary devices and percentage of time spent in a wheelchair. It is an ordinal measure that includes descriptions of scores: 0 – absence of functional or ambulation impairment; 5 – maximum impairment, more than 50% of time spent in a wheelchair.
4. Modified Ashworth Scale (MAS) [9]: a qualitative instrument with ordinal measurements represented by descriptions of scores. Assessment is based on the passive motion of the upper limbs, with the individuals remaining at rest. The spasticity-induced resistance of the quadriceps femoris, triceps surae and hip adductors in the supine position is scored from 0 to 5.
5. Motor Assessment Scale [10]: this scale allows assessing the ability to remain sitting; a higher score indicates a greater ability.
6. Ambulation Index (DeLuca): described by Hauser (1983) and revised by Chiaravalloti and DeLuca [11]. This index allows characterising and standardising the stage of disease regarding ambulation and need for a wheelchair. A higher score indicates a poorer ability to ambulate.

### Statistical tests

Descriptive measures are expressed as the means and standard deviations when appropriate, and as medians and interquartile ranges otherwise. Data normality was assessed using the Anderson-Darling test. The paired samples t-test was used to compare between time points when distribution was normal; otherwise, the Wilcoxon signed-rank test was used. Groups (2007 and 2017) within the cohort were compared by means of the independent samples t-test or the Mann-Whitney U test when the data distribution was non-normal. Categorical variables were analysed using Fisher’s test. Correlations were investigated with Pearson’s or Spearman’s tests.

## Results

The sample consisted of 47 individuals, including 31 females and 16 males. A total of 25 participants were illiterate, 14 had attended approximately 4 years of formal schooling, and four had attended 8 years. Only one participant had completed higher education, and three did not answer this question. Twenty-nine participants received the state-provided Continued Assistance Benefit corresponding to the equivalent of the minimum wage. The remainder of the sample had no source of income but were instead dependent on relatives. All the data are described based on the comparison of the data collected from the same individuals in 2007 and 2017.

The mean age of the sample was 36.87 ± 12.50 in 2007 and 47.21 ± 12.42 years old in 2017. There was no significant difference (*p* > 0.05) in the mean age between females and males. On the last assessment, the age of the participants varied from 22 to 81 years old. The median age at onset of symptoms was 1 year old, with variations for the first and third quartiles being 1 and 2 years, respectively. The mean age at loss of ambulation was 10.78 ± 5.5 years old. Only eight of the participants had preserved hand grip strength as assessed with the Jamar® dynamometer (Table 1). Only two participants were still able to walk with some aid.

**Table 1:** Participants’ characteristics and mean and median ages; comparison of values along the analysed 10-year period by means of the Mann-Whitney U test

Variables	First visit 2007	Second visit 2017	*p*-value
*Number of patients*	47	47	–
Male	16	16	–
Female	31	31	–
*Age (years)*	36.87 ± 12.50	47.21 ± 12.42	–
Male	36.67 ± 9.90	46.81 ± 9.89	–
Female	36.96 ± 13.81	47.45 ± 13.70	–
*Age at onset (years)*	1 [1.00; 2.00]	1 [1.00; 2.00]	–
*Age of gait loss*	10.00 [7.00; 15.00]	10.00 [7.00; 15.00]	–
*Age of manual function loss*	–	38.00 [20.00; 41.50]	–
*Barthel Índice*	39.00 [24.50; 51.50]	22.00 [18.00;33.50]	< 0.001^a^
*Grip strength R*	3.00 [0.00; 9.00]	0.00 [0.00; 0.00]	< 0.001^a^
*Grip strength L*	2.00 [0.00; 7.00]	0.00 [0.00; 0.00]	< 0.001^a^
*SPRS*	40.50 [38.00;43.75]	41.00 [38.00;44.00]	0.195
*Deluca*	9.00 [8.00; 9.00]	9.00 [9.00;9.00]	0.982
*Smith*	1.00 [1.00; 2.00]	1.00 [1.00; 1.00]	0.257
*Fink Scale*	5.00 [4.0; 5.0]	5.00 [5.0; 5.0]	0.998

Numbers in brackets represent the interquartile range

^a^Mann-Whitney U test, *p*-value were calculated between first and second visit

The Anderson-Darling normality test evidenced that the following variables had non-normal distribution: age in 2007 and 2017; age at loss of hand function; MBI; and SPRS. For this reason, the median values obtained in 2007 and 2017 were compared by means of the Mann-Whitney U test.

The median score on the MBI differed significantly (*p* < 0.001), indicating that the level of dependence increased over time. The U test did not detect significant differences in the median scores on SPRS and FHSPRS from 2007 to 2017 (Table 1). These scales were used to assess ambulation ability and the influence of spasticity on ambulation. In 2007, 68.08% of the patients with Spoan syndrome were already confined to a wheelchair; 10 years later, almost all of them (97.87%) were wheelchair-bound.

The Motor Assessment Scale results showed that in 2017, 82.97% of the participants could sit only with support. The results for the Modified Ashworth Scale for that year showed that 34.04% of the sample exhibited grade 1 and 14.89% grade 4 spasticity. The unpaired t-test was used to compare means between subgroups (Table 1). The differences between medians were significant for both right- and left-hand grip strength. The median value decreased from 3.33 to 0 kgf on the right hand and from 1.66 to 0 kgf on the left from 2007 to 2017 (Table 1).

**Fig. 1:**
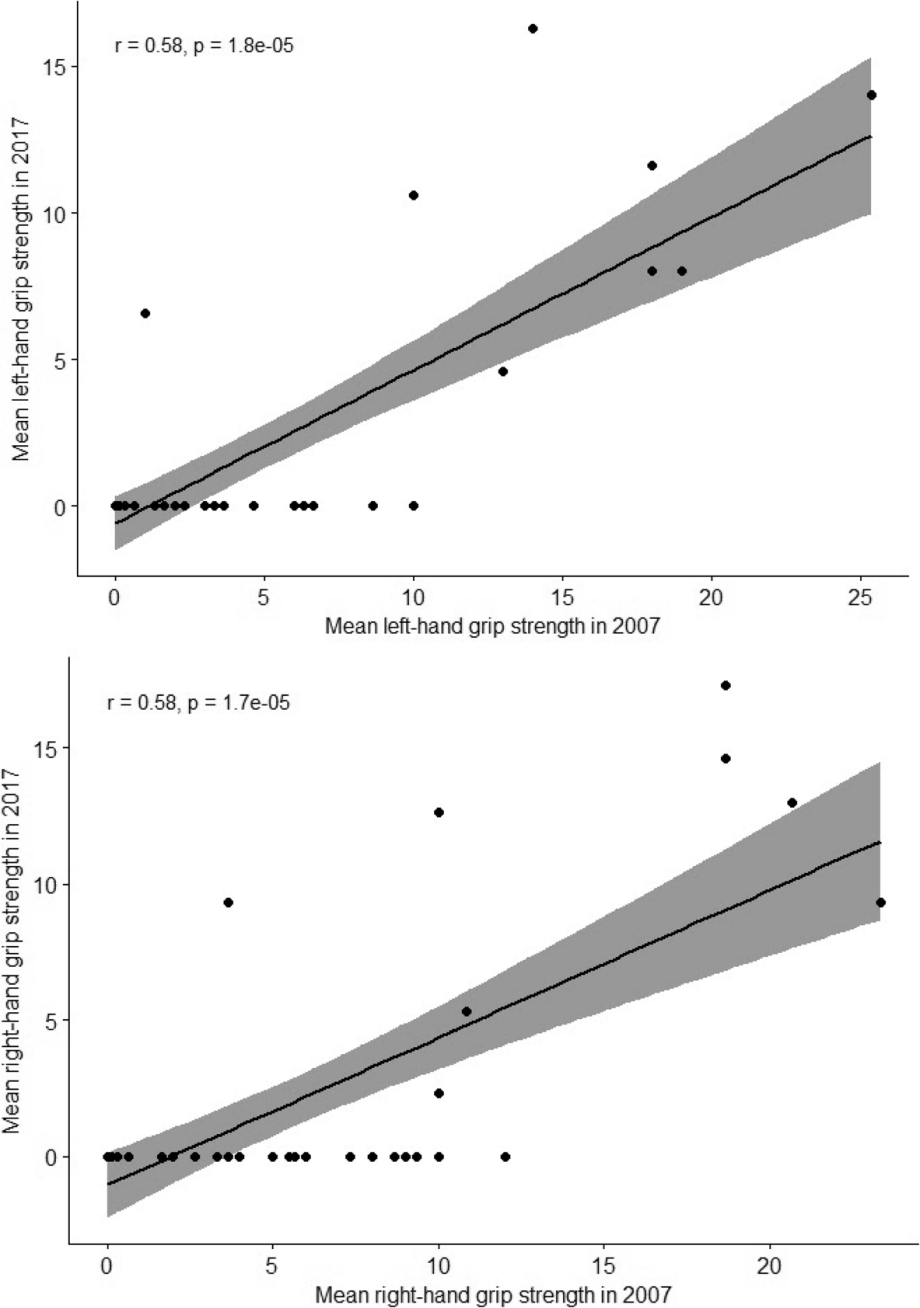
Correlations between the mean right- and left-hand grip strength in 2007 and 2017

At the time of the first assessment in 2007, 14 participants had already fully lost their hand grip strength. Ten years later, this finding was detected in an additional 25 patients, and only eight still exhibited some degree of hand grip strength, as assessed with the Jamar® hydraulic dynamometer; the right hand was the dominant one for all of these cases. Figure 1 depicts the correlations between right- and left-hand grip strength in 2007 and 2017.

Relative to loss of hand function in association with dependence for feeding, among the 26 participants who fully lost their hand function, 15 were total dependent for feeding, while 11 were still able to handle eating utensils, albeit with constant assistance; the scores on the MBI domain feeding ranged from 0 to 2. Relative to the other 21 participants, the scores on the MBI domain feeding ranged from 5 to 8; six were able to eat with supervision, while 15 were able to eat independently.

Non-parametric correlation analysis (Spearman’s correlation) between the score on the MBI and the investigated variables evidenced statistical significance for age (*p* < 0.001) and right- and left-hand grip strength (*p* = 0.042 and *p* = 0.021, respectively). Statistical significance was not evidenced for the remainder of the variables, including age at onset of symptoms (*p* = 0.634), SPRS (*p* = 0.832), age at loss of ambulation (*p* = 0.664) and age at loss of hand function (*p* = 0.118).

**Fig. 2:**
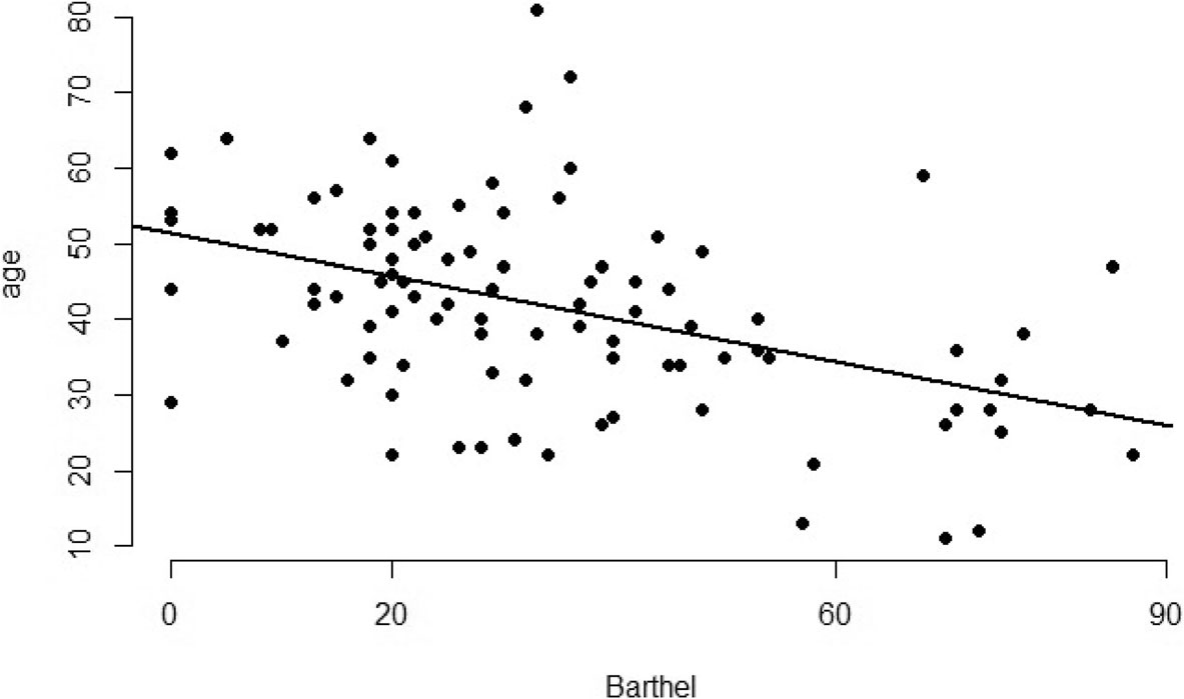
Linear regression evidencing the relationship between age and MBI(00–20: Total Dependence; 21–60: Severe Dependence; 61–90: Moderate Dependence; 91–99: Slight Dependence; 100: Independence) [6]

Since we found correlation between variation in age and MBI, we fit a simple linear regression model to investigate the effect of the level of loss of independence over time. The equation that represents the relationship between these two variables is y = 52.37–0.30x, where y is variation in age, and x the MBI. The *p*-value for coefficients β_0_ and β_1_ was < 0.05, i.e., statistically significant. Figure 2 depicts the regression line fitted to the data.

**Fig. 3:**
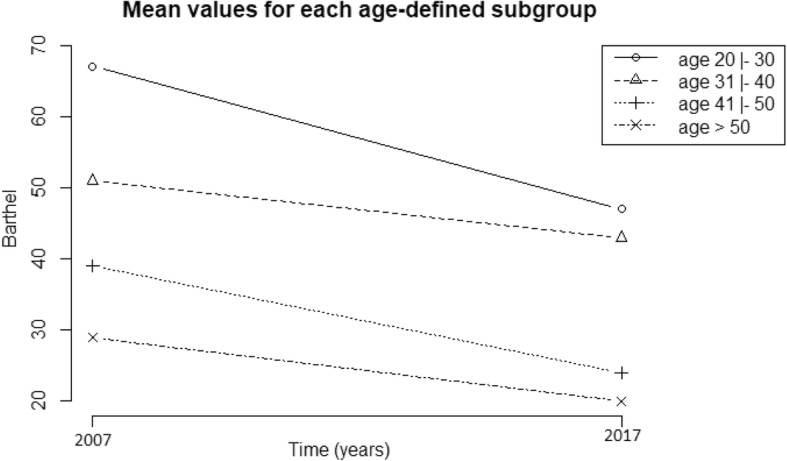
Profile plot showing the progression of the median scores on the Modified Barthel Index comparatively assessed from 2007 to 2017, subdivided by age range

In 2017, 87.23% of the participants exhibited severe or total dependence according to the score on the MBI. Since the level of dependence increased with age, we performed an analysis of profile plots for a closer look into age ranges, as shown in Fig. 3. More than half of the participants (58.33%) within the age range of 31 to 40 years old (25.53% of the sample) were able to perform transfers and self-care activities, albeit with some degree of dependence. In the group aged 41 to 50 years old, 23.40% exhibited total dependence. However, half of them were still able to eat by themselves with spoons and under continued supervision; some had only their bladder and bowel control preserved and one case exhibited major losses in bathing, dressing and transfer ability (Fig. 3). Most of the participants over 50 years old (44.68%) exhibited total dependence. Only three patients were still able to participate at some level in their feeding.

## Discussion

The present study is the first longitudinal study of a complex and recessive form of spastic paraplegia prevalent in the semi-arid Northeast region of Brazil. Longitudinal studies are indicated to describe the progression rates in HSP and determine whether the nonlinear increase in disease severity in later disease stages reflects true disease evolution in HSP or represents an artefact of the cross-sectional study design [12]. To our knowledge, only two studies evaluated cross-sectional and longitudinal disease progression in HSP and most of the participants had the dominant forms [12, 13].

Our analysis allows asserting that Spoan individuals exhibited slight dependence until age 35. The greatest losses occurred from ages 35 to 41, and starting at 50, practically all patients become completely dependent. In fact, age is the main predictor factor of impaired motor function among Spoan syndrome patients. Among the 47 reassessed individuals, 14 had already lost their hand grip strength on the first assessment in 2007. Of the remaining individuals, 25 completely lost their hand grip strength over the following 10 years, and eight exhibited partial loss. All the participants needed to use a wheelchair, and only two were able to walk with some aid.

The loss of hand grip strength over the 10-year period was associated with higher levels of dependence. For the eight participants with preserved hand grip strength, the values obtained with the Jamar® dynamometer were less than half of the expected values. Unexpectedly, 21 of the participants with no hand grip strength were able to feed themselves somehow without any adaptation of the utensils.

The decline in the scores on the functional classification scale reflects the progressive nature of the disease with a marked axonal and sensory-motor neuropathy. Amorim et al. (2014) [14] conducted a study on nerve, motor and sensory conduction that involved 27 cases of people with Spoan syndrome aged between 4 and 58 years, of which 20 were women. All cases had severe neuropathic signs and demonstrated a deficit of force and distal atrophy, and 58% of them had deformities in the feet and spine. The deep reflexes of the upper limbs were exalted in 92% of the cases, and the patellar reflex in 63%; being the Aquileu reflex absent in all patients. No correlation was observed between age and conduction velocity, latency and amplitude of median and ulnar nerves. There was a reduction in conduction velocities in the median and ulnar nerves by 50 and 41%, respectively. The motor latencies of the axillary and femoral nerves were normal in all cases. Changes in driving speed are probably due to the loss of rapidly conducting nerve fibers 20 [14].

Schüle and collaborators (2016) [12] evaluated 608 cases of HSPs and pointed out that the age of onset and disease duration showed the strongest effect on disease severity, whereby late age of onset and longer disease duration were associated with higher SPRS scores. In our cohort, the first symptoms, such as optic atrophy and spastic paraparesis, appear before age five and most frequently during the first year of life. In fact, it was not found a significant variation in the age at onset in Spoan syndrome. For this reason, no correlation was found between age at onset of symptoms and motor impairment or between motor impairment and sex, as described in follow-up studies of other rare genetic diseases [12, 13, 15, 16].

Data from larger cohorts using measures validated for HSP are essentially missing in the literature [15]. In the present study, no significant difference was found on Schüle’s SPRS, Fink’s FHSPRS or the Ambulation Index revised by Chiaravalloti and DeLuca between 2007 and 2017. The participants’ ambulation was already highly impaired at the first assessment in 2007, as 70.21% of the sample spent more than 50% of their time in a wheelchair, and only three were able to walk. In 2017, 45 individuals (97.87%) had completely lost their ambulation ability and spent their entire days in a wheelchair. A high proportion of affected individuals had minimum or maximum scores on the scales, which might reduce variability of data and explain the outcomes.

Although the patients’ life expectancy was not investigated using suitable methods, it does not seem to decrease, since six participants were 60 years old or older, and one was 81 years old. Regarding causes of death, no repeated respiratory problems (pneumonia) were detected, as in other studies on rare genetic diseases [16, 17]. Therefore, Spoan syndrome is different from conditions such as Duchenne muscular dystrophy, in which the life expectancy is limited to two or three decades as a function of cardiac and respiratory involvement [18], and amyotrophic lateral sclerosis, with a life expectancy of 2 to 5 years after diagnosis [19, 20]. Some patients exhibited dysphagia, none required a feeding tube. The causes of death bore no relationship to the disease, and included cancer, infarction, stroke and congenital heart disease.

One of the limitations of the present study concerns the number and variety of the instruments applied at the time Spoan syndrome was first described, which restricted the number of predictive variables. For instance, psychological aspects of patients and their quality of life were not investigated on the earlier assessment. Moreover, it was not possible to establish whether rehabilitation and assistive technology might influence the speed of motor function impairment and the development of deformities, because seven individuals were only attending physiotherapy once a week. Residents of the state of Rio Grande do Norte still lack access to specialised rehabilitation services.

## Conclusions

For the first time, age was described as a predictor factor of the clinical outcome in Spoan syndrome. These findings might be relevant for determining the prognosis as well as the suitable treatment, rehabilitation and assistive technology for these individuals.

## Data Availability

The datasets used and/or analysed during the current study are available from the corresponding author on reasonable request.

## Abbreviations

FHSPRS: Functional Hereditary Spastic Paraplegia Rating Scale
kgf: Kilogram-force
*KLC2*: Kinesin light chain 2 gene
MAS: Modified Ashworth Scale
MBI: Modified Barthel Index
*p*-value: Probability value
Spoan syndrome: Spastic Paraplegia Optic Atrophy and Neuropathy
SPRS: Spastic Paraplegia Rating Scale
